# SIRT5 mediates the pro-osteogenic effects of estrogen through FDX1 demalonylation and cuproptosis inhibition in mesenchymal stem cells

**DOI:** 10.1016/j.gendis.2026.102145

**Published:** 2026-03-17

**Authors:** Fanglin Ye, Dongmei He, Wenting Liu, Jie Cai, Aihua Ye, Zhenghao Xu, Wenge He, Yuxi Su, Junyi Liao, Baicheng He

**Affiliations:** a Department of Pharmacology, School of Pharmacy, Chongqing Medical University, Chongqing 400016, China; b Key Laboratory of Biochemistry and Molecular Pharmacology of Chongqing, Chongqing Medical University, Chongqing 400016, China; c Department of Orthopedics, Children’s Hospital of Chongqing Medical University, Ministry of Education Key Laboratory of Child Development and Disorders, Chongqing 400014, China; d Department of Bone and Soft Tissue Oncology, Chongqing University Cancer Hospital, Chongqing 400030, China; e Department of Orthropetics, the First Affiliated Hospital of Chongqing Medical University, Chongqing 400016, China

**Keywords:** Cuproptosis, Demalonylation, Ferredoxin 1, Osteoporosis, Sirtuin 5

## Abstract

Osteoporosis, a common orthopedic disease predominantly caused by estrogen deficiency in postmenopausal women, continues to pose a significant public health challenge due to the poorly understood molecular mechanisms. While cuproptosis has been implicated in various pathological conditions, its concrete role in the pathogenesis of osteoporosis remains unknown. Equally ambiguous remains the functional role of Sirtuin 5 (SIRT5), a mitochondrial deacylase with well-characterized involvement in aging and bone formation, in estrogen deficiency-associated osteoporosis. In the present study, we identified a novel potential Estrogen/SIRT5/Ferredoxin 1 regulatory axis that modulates both cuproptosis and the lineage commitment of mesenchymal stem cells. Using an ovariectomized mouse model, we observed that serum copper levels were reduced, whereas copper accumulation was elevated in bone tissue. Estrogen deficiency down-regulated SIRT5 expression, promoted cuproptosis, and induced obvious bone loss. Cuproptosis directly impaired the osteogenic differentiation in mesenchymal stem cells, while SIRT5 overexpression partially rescued this lineage commitment defect. Mechanistically, we showed that estrogen up-regulated SIRT5 expression, which in turn mediated Ferredoxin 1 demalonylation and enhanced its lysosomal degradation. This dual regulatory mechanism may effectively suppress cuproptosis and restore the osteogenic potential of mesenchymal stem cells. Our findings suggest that the novel Estrogen/SIRT5/FDX1 axis may function as a key regulator of bone homeostasis, and identify SIRT5 as a potential therapeutic candidate for postmenopausal osteoporosis, likely through its capacity to reduce the cuproptosis-like features of mesenchymal stem cells.

## Introduction

Osteoporosis is a prevalent skeletal disorder characterized by progressive bone loss and deterioration of bone microarchitecture.^1^, ^2^, ^3^ Estrogen plays an essential role in maintaining healthy bone metabolism,^3^ and its deficiency, particularly following menopause, is a major cause of osteoporosis.^4^ While estrogen can directly influence osteoblast-osteoclast homeostasis by inhibiting osteoblast apoptosis and enhancing their functional capacity,^3^^,^^5^ the concrete underlying molecular mechanisms by which estrogen modulates bone metabolism remain incompletely elucidated. The estrogen-mediated protection against bone loss is primarily mediated via estrogen receptor α (ERα) signaling.^6^ However, the precise downstream mechanisms remain poorly defined.^7^ A deeper understanding of this biological process is crucial for developing more effective therapeutic strategies for postmenopausal osteoporosis.

Recent studies have highlighted a strong association between the sirtuin family of protein deacetylases/deacylases and osteoporosis 8, 9, 10, 11. Although Sirtuin1, Sirtuin3, Sirtuin6, and Sirtuin7 have been implicated in the onset and progression of osteoporosis 12, 13, 14, 15, the role of Sirtuin2, Sirtuin4, and Sirtuin5 (SIRT5) in regulating bone metabolism remains unknown. Among the seven sirtuin isoforms, SIRT5 is a lysine deacetylase primarily located in mitochondria, known for catalyzing the desuccinylation, demalonylation, and deacetylation of lysine residues.^16^^,^^17^ SIRT5 participates in various physiological processes, including cellular metabolism and detoxification of reactive oxygen species.^16^ For instance, SIRT5 can attenuate compression-induced intervertebral disc degeneration via desuccinylation of apoptosis-inducing factor mitochondria-associated 1 (AIFM1).^18^ Emerging evidence suggests a role for SIRT5 in bone health: Sirt5 knockout exacerbates bone loss in ovariectomized (OVX) mouse models,^19^ and our previous work has demonstrated that SIRT5 enhances the osteo-inductive potential of bone morphogenetic protein 9 (BMP9).^20^ Despite these observations, the specific role of SIRT5 in estrogen deficiency-induced osteoporosis and the associated precise mechanisms remain undefined.

Copper, an essential trace element, plays an indispensable role in cellular growth and metabolism, with a significant portion distributed in muscle and bone tissue 21, 22, 23. Copper is implicated in the regulation of bone metabolism and skeletal development 24, 25, 26. While low concentrations of copper are vital for these processes 27, 28, 29, high concentrations of copper can induce cytotoxicity in osteoblasts.^25^^,^^30^^,^^31^ Cuproptosis, a form of cell death triggered by excessive copper accumulation,^32^ has garnered attention for its potential contribution to various orthopedic diseases, including osteoporosis 33, 34, 35. Ferredoxin 1 (FDX1), as an upstream regulator of protein lipoylation, is critical for triggering cuproptosis by reducing Cu^2+^ to the more toxic Cu^1+^.^32^ It has also been identified as a direct target of elesclomol, a cuproptosis inducer.^36^ FDX1 expression is up-regulated in degenerative intervertebral discs, atherosclerosis, and in patients with arthritis who have a poor prognosis.^37^^,^^38^ However, the specific involvement and regulatory mechanisms of cuproptosis in the pathogenesis of osteoporosis remain unclear.

Given the critical roles of estrogen in regulating bone metabolism, the emerging importance of SIRT5 in age-related diseases, and the significance of copper for bone homeostasis, we hypothesized a mechanistic link between these factors in osteoporosis. In this study, we investigated whether the Estrogen/SIRT5/FDX1 axis serves as a key regulator of cuproptosis and osteogenic differentiation in mesenchymal stem cells.

## Materials and methods

### Cell culture and reagents

C3H10T1/2 and HEK293 cells were ordered from the American Type Culture Collection (Manassas, Virginia, USA). The medium used for cell culture contained 10% fetal bovine serum, 100 μg/mL streptomycin, and 100 U/mL penicillin. Cells were incubated at 37 °C and 5% CO_2_. Elesclomol (HY-12040), MC3138 (HY-160818), BafA1(HY-100558), and MG132(HY-13259) were purchased from MCE (Shanghai, China). Cupric chloride (751944) was ordered from Sigma–Aldrich (Shanghai, China). Tetrathiomolybdate (TTM, A828261) was ordered from MACKLIN (Shanghai, China), and tamoxifen (S48264) and estradiol (S30633) were bought from Yuanye Biotechnology (Shanghai, China). Recombinant adenovirus and adeno-associated virus for Sirt5 and Fdx1 were ordered from Obio Technology (Shanghai, China). Primary antibody against β-actin (AC026), RUNX2 (A2581), and FDX1 (A20895) were ordered from ABclonal (Wuhan, China); SIRT5 (5122-1-AP) and Ubiquitin (10201-2-AP) were ordered from Proteintech (Wuhan, China); Malonyllysine (PTM-901) was ordered from PTM Biolabs (Hongzhou, China); acetyl Lysine (ab80178) was ordered from Abcam (Shanghai, China); ATP7A (TD8506) and CTR1 (T510261F) were ordered from Abmart (Shanghai, China); OPN (sc-10593) was ordered from Santa Cruz Biotechnology (Shanghai, China).

### Mouse OVX model establishment and histochemical analysis

Female C57BL/6 mice (8 weeks old, 16 g) were purchased from the experimental animal center of Chongqing Medical University and randomly divided into five groups: Sham, OVX, OVX + High copper, OVX + TTM, and OVX + MC3138. This animal experiment was approved by the Institutional Animal Care and Utilization Committee of Chongqing Medical University (IACUC-CQMU-2024-0617). Briefly, ovariectomy was performed as follows: two dorsolateral incisions were made to locate and exteriorize two ovaries. Upon removal of the ovaries, the peritoneal cavity and skin were closed. The sham group underwent sham surgery only. One week after surgery, the mice in the OVX + High copper group were fed with a high-copper diet (M22042002, BIOPIKE) for 8 consecutive weeks. Mice in the OVX + MC3138 group received intraperitoneal injections of MC3138 (a SIRT5 activator, 150 mg/kg/day) for 8 weeks.^39^ Mice in the OVX + TTM group were administered intraperitoneal injections of TTM at a daily dose of 10 mg/kg, three times per week for 8 weeks.^40^ At the experimental endpoint, all mice were euthanized. Femoral samples were collected and fixed with 4% paraformaldehyde. Subsequently, the fixed samples were decalcified in 10% ethylenediaminetetraacetic acid and then processed into paraffin sections.

### RNA extraction, reverse transcription (RT), and quantitative PCR analysis

Total RNA was extracted from samples using the RNA-Quick Purification Kit (RN001, ES Science) following the protocol provided by the manufacturer. After the reverse transcription reaction, complementary deoxyribonucleic acid (cDNA) was mixed with 2 × SYBR Green qPCR Master Mix (SMK-Q002, Saimike), and quantitative PCR was performed on a Bio-Rad CFX apparatus using the following protocol: pre-denaturation at 95 °C for 3 min, then denaturation at 95 °C for 5 s, annealing at 60 °C for 30 s, and extension at 72 °C for 40 cycles. The relative expression of mRNA levels of the target gene was calculated using the 2^−ΔΔCt^ method and normalized to the housekeeping gene β-actin. The specific primer sequences used in this study are listed in Table 1.

**Table 1:** Primers used in this study.

Gene	Primer	Sequence (5′-3′)
*β-actin*	F	CCACCATGTACCCAGGCATT
	R	CGGACTCATCGTACTCCTGC
*Runx2*	F	GCCAATCCCTAAGTGTGGCT
	R	AACAGAGAGCGAGGGGGTAT
*Opn*	F	TGCACCCAGATCCTATAGCC
	R	CTCCATCGTCATCATCATCG
*Sirt5*	F	ACTCTTCCTGAAGCCCTTGC
	R	TTGGGGCTTGAAGGGTGTTT
*Atp7a*	F	ACCCCGAGTGATAGCAGAGT
	R	GCTTTGTTAGTTGCCAGGGC
*Ctr1*	F	GTGGTAACCCTGGTGCTCTC
	R	CCTACGGGCCTTGGTTCTTT
*Fdx1*	F	CCAAGGGGAAAATTGGCGAC
	R	TCCAAAAGCCAGGTCAAGCA
*Lias*	F	AATTGCAGAGTGGGGTCTGG
	R	CAGAGCCACCTTCTCCACTG
*Dlat*	F	GTGCTGTTGGTACGGAAGGA
	R	GCAACACTGACGTCAACCAC

(F: forward, R: reverse).

### Western blotting analysis

Protein samples were initially separated via 10% or 12.5% sodium dodecyl sulfate polyacrylamide gel electrophoresis (SDS-PAGE; PG112; PG113, Epizyme Biotech) and then transferred onto polyvinylidene fluoride (PVDF) membrane (PR05509, Merck). Membranes were blocked with protein-free rapid blocking buffer (PS108P, Epizyme Biotech) before overnight incubation with primary antibodies. After washing with Tris-buffered saline-Tween 20 three times, membranes were incubated with BeyoWB™ HRP-labeled Goat Anti-Rabbit I (P0948, Beyotime) or BeyoWB™ HRP-labeled Goat Anti-Mouse (P0946, Beyotime) at room temperature for 1 h. Finally, blots were visualized using a chemiluminescent detection kit (160072, Saimike Biotech), and images were acquired using the Bio-Rad ChemiDoc XRS + system (Bio-Rad, Hercules, California, USA).

### Immunofluorescent staining

After deparaffinization, rehydration, and antigen retrieval, sections were rinsed and incubated in a hydrogen peroxide solution. After air-drying, sections were blocked with bovine serum albumin solution (A8010, Solarbio). Primary antibodies were then added, and sections were incubated at 4 °C overnight. After washing with phosphate-buffered saline (PBS), sections were incubated with 594 goat anti-rabbit (Y6107S, UElandy) or 488 goat anti-mouse (Y6104S, UElandy) at room temperature for 1 h. After washing with 1 × PBS (G4202-100 ML, Servicebio), sections were counterstained with 4′,6-diamidino-2-phenylindole (C1006, Beyotime). Fluorescent images were taken with a Leica TCS SP5 Confocal Laser Scanning Microscope (Leica, Wetzlar, Germany).

### Immunoprecipitation assay

Cells were lysed in lysis buffer supplemented with protease and phosphatase inhibitors (B15001, Bimake). Cell lysates were pre-cleared with Protein A/G magnetic beads (P2108, Beyotime) before overnight incubation at 4 °C with primary antibodies against FDX1, malonyllysine, acetyllysine, ubiquitin, SIRT5, or rabbit IgG. The beads were meticulously washed twice with RIPA lysis buffer (QS0006, Saimike), followed by boiling for 10 min in RIPA lysis buffer to elute the proteins. Finally, Western blotting analysis was performed for subsequent detection.

### Alkaline phosphatase (ALP) staining

ALP Kit (C3206, Beyotime) was used to determine the ALP activities in C3H10T1/2 cells on days 5 and 7 post-induction. Briefly, cells were fixed with paraformaldehyde at room temperature for 20 min, followed by washing with PBS 3 times (5 min per wash). Subsequently, 200 μL of the assay working solution was added to each well of the 24-well plate, and cells were incubated at room temperature for 15–30 min. After two washes with distilled water, the plates were scanned, and bright-field images were acquired using an Olympus IX53 microscope (Olympus Corporation, Tokyo, Japan). The ALP activity was quantified using ImageJ software (National Institutes of Health, Bethesda, Maryland, USA).

### Mineralization assay

Cells were maintained in complete DMEM with osteogenic induction cocktail (10 nM dexamethasone, 50 μg/mL ascorbic acid, and 10 mM β-glycerophosphate). On days 14 or 21 post-treatment, mineralized nodules were visualized with 0.4% alizarin red S (A5533-25G, Sigma–Aldrich). The plates were scanned, and bright-field images were obtained using a microscope (IX53, Olympus, Japan). Quantification of mineralized nodules was subsequently performed using ImageJ software (National Institutes of Health, Bethesda, Maryland, USA).

### Micro-computed tomography

Femurs from mice were scanned using a micro-computed tomography system (Bruker SKYSCAN 1276; Bruker Corporation, Billerica, Maryland, USA). Three-dimensional images were reconstructed, and bone morphometric parameters were also quantified, including bone mineral density (BMD), bone volume fraction (BV/TV), trabecular number (Tb. N.), and trabecular thickness (Tb. Th.).

### Cellular immunofluorescent staining

Cell slides were fixed with 4% paraformaldehyde at room temperature for 15 min, treated with QuickBlock™ blocking buffer (Beyotime Biotechnology, China) for 30 min, and rinsed twice with PBS. Slides were then incubated overnight with primary antibodies. Following two PBS washes, slides were incubated with Alexa Fluor 488-labeled goat anti-mouse (Servicebio GB25301, 1:200) or Alexa Fluor 594-labeled goat anti-rabbit (Servicebio GB28301, 1:200) at room temperature in the dark for 1 h. Finally, slides were counterstained with 4′,6-diamidino-2-phenylindole (DAPI, C1006, Beyotime) at room temperature in the dark for 10 min. Fluorescence images were taken using a Confocal Microscope (Leica TCS SP8), and fluorescence intensity was quantified with ImageJ software (National Institutes of Health, Bethesda, Maryland, USA).

### Mito-tracker staining assay

Cells were seeded in 15 mm dishes and treated with elesclomol, AdSirt5, and/or AdFdx1 to assess the number of bioactive mitochondria. Mito-Tracker Red (C1032, Beyotime) was added to the cells and incubated at 37 °C for 30 min. Nuclear location was subsequently performed using DAPI reagent (C1006, Beyotime). Images were taken using a Leica TCS SP5 Confocal Microscope (Leica, Wetzlar, Germany).

### Transmission electron microscopy analysis

Cells were initially detached by trypsin (SMK0104, Saimike) for 1 min and collected into a centrifuge tube, and centrifuged at 1000 rpm (4 °C) for 5 min. The supernatant was discarded, and cells were gently resuspended in 0.5% glutaraldehyde fixing solution (G916054, MACKLIN). The resuspended cells were incubated at 4 °C for 5 min and centrifuged at 12,000 rpm (4 °C) for 10 min. After careful removal of the supernatant, 2.5% glutaraldehyde fixing solution was added for further fixation. Finally, samples were then processed and imaged via transmission electron microscopy.

### Mitochondrial membrane potential assay

The mitochondrial membrane potential was evaluated using the JC-1 Mitochondrial Membrane Potential Assay Kit (C2006, Beyotime) according to the manufacturer’s instructions. Briefly, JC-1 dye working solution was added to cultured cells and incubated at 37 °C in the dark for 30 min. Cells were subsequently washed with serum-free medium. Finally, fluorescent images were acquired using a confocal microscope (Leica TCS SP8).

### Lysosome staining assay

Cells were seeded in 15 mm glass-bottom cell culture dishes and treated with dimethyl sulfoxide (DMSO; SB0061, Saimike) or estradiol (S30633) for 24 h before lysosome staining. Cells were incubated with 50 nM LysoTracker Red at 37 °C for 60 min. Fluorescent images were acquired using a confocal microscope (Leica TCS SP8).

### Copper content assay

Copper levels in cells, culture medium, and serum were quantified using copper colorimetric assay kits (E-BC-K300-M, Elabscience) following the manufacturer’s protocol. Briefly, supernatants from tissues or cells were incubated with assay buffers at 37 °C for 5 min. Absorbance was measured at 580 nm using a multifunctional microplate reader (ReadMax 1500, Flash spectrum Biological Technology). Copper concentrations were calculated based on standard curves generated according to the manufacturer’s instructions.

### Protein docking analysis

Crystal structures of SIRT5 and FDX1 were obtained from the RCSB Protein Data Bank (https://www.rcsb.org/). These structures were then submitted to ClusPro (https://cluspro.org/home.php) for protein–protein docking. Following clustering and energy minimization, a panel of protein–protein complex was generated, including interactions driven by electrostatics, van der Waals forces, and hydrophobic effects. For comprehensive analysis, the top-ranked model from the balanced scoring list was selected. LigPlot+2.2.4 was used to identify functional residues involved in hydrogen bonding interactions, salt bridges, and hydrophobic interactions. PyMol 2.2.0 was utilized to visualize the docking conformation. SIRT5 was represented as a pistachio cartoon, FDX1 as a cyan cartoon, and their binding interfaces were highlighted. Moreover, the Prodigy tool (https://bianca.science.uu.nl/prodigy/) was employed to calculate the binding free energy of the SIRT5–FDX1 complex.

### Statistical analysis

Data were presented as mean ± standard error of the mean. All assays were performed independently with three biological replicates. Statistical analysis was conducted using GraphPad Prism 9.5.0 software. The difference between the two groups was assessed for statistical significance using the Student’s *t*-test, while one-way analysis of variance (ANOVA) was employed for comparisons across three or more groups. The *P*-value < 0.05 was considered statistically significant.

## Results

### Effects of OVX or estradiol on SIRT5 expression and cuproptosis markers

**Figure 1:**
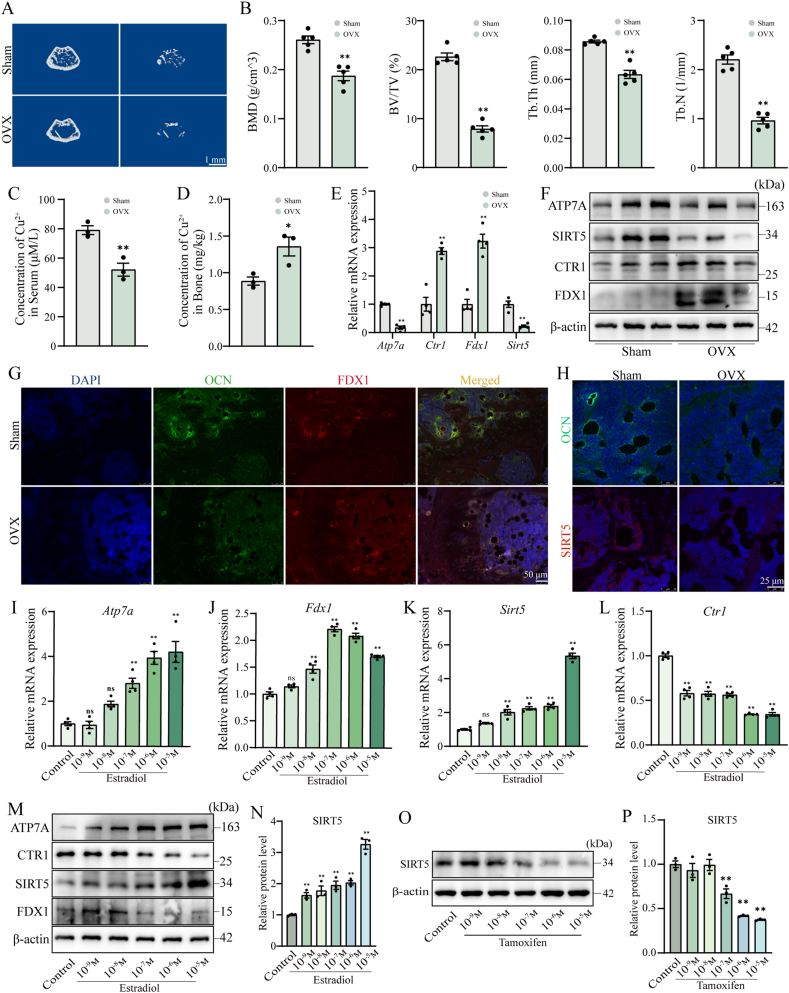
Effects of ovariectomy or estradiol on SIRT5 expression and cuproptosis markers. **(A)** Micro-CT analysis results of the mice’s distal femur (scale bar: 1 mm). **(B)** Quantitative results of micro-CT analysis show the bone mineral density (BMD), the ratio of the bone volume to the total volume (BV/TV), the number of trabeculae (Tb.N), and the trabecular thickness (Tb.Th) (*n* = 5). **(C, D)** Serum and bone tissue levels of Cu^2+^ in mice (*n* = 3). **(E, F)** Quantitative PCR and Western blotting assay results show the expression of FDX1, SIRT5, CTR1, and ATP7A. **(G, H)** Representative ICC images show the expression of FDX1 and SIRT5 in mice femur (scale bar = 50 μm for 1G or 25 μm for 1H). **(I**–**L)** Quantitative PCR assay results show the mRNA expression levels of *Atp7a*, *Fdx1*, *Sirt5*, and *Ctr1* in C3H10T1/2 cells. **(M)** Western blotting assay results show the protein levels of FDX1, SIRT5, CTR1, and ATP7A in C3H10T1/2 cells treated with estradiol. **(N)** Quantitative results of Western blotting assay show the protein levels of SIRT5 in C3H10T1/2 cells treated with estradiol. **(O)** Western blotting assay results show the protein levels of SIRT5 in C3H10T1/2 cells treated with tamoxifen. **(P)** Quantitative results of Western blotting assay show the protein levels of SIRT5 in C3H10T1/2 cells treated with tamoxifen. ^*ns*^*P* > 0.05, *∗P* < 0.05, and *∗∗P* < 0.01.

To determine the relationship between estrogen deficiency-induced osteoporosis and SIRT5 and/or cuproptosis, we first established the OVX mouse model. Micro-CT three-dimensional reconstruction and quantitative analysis confirmed the successful induction of osteoporosis in OVX mice (Fig. 1A and B). Subsequently, serum and bone tissue copper levels were measured in the sham-operated group and OVX group. Results revealed that serum copper levels were decreased in OVX mice, while copper levels in bone tissue were increased (Fig. 1C and D). SIRT5 and other cuproptosis-related markers were assessed in bone tissue. Quantitative PCR and Western blotting results showed that FDX1 and CTR1 were increased, while SIRT5 and ATP7A were decreased in the bone mass from OVX mice (Fig. 1E and F). Immunofluorescent staining results exhibited that both OCN and SIRT5 were decreased, but FDX1 was significantly increased (Fig. 1G and H). Consistent observations were obtained with immunohistochemical staining (data not shown). To further validate these results *in vitro*, we treated cells with estrogen. Quantitative PCR and Western blotting results showed that estrogen concentration-dependently up-regulated SIRT5 and ATP7A, while reducing Ctr1 (Fig. 1I–L). Concomitantly, estrogen elevated FDX1 protein level (Fig. 1M and N). On the contrary, SIRT5 protein level was reduced by tamoxifen concentration-dependently in C3H10T1/2 cells (Fig. 1O and P). These results suggest that estrogen deficiency-induced osteoporosis may be associated with the reduced SIRT5 expression and activation of cuproptosis.

### Effects of copper or SIRT5 activator on osteoporosis in OVX mice

**Figure 2:**
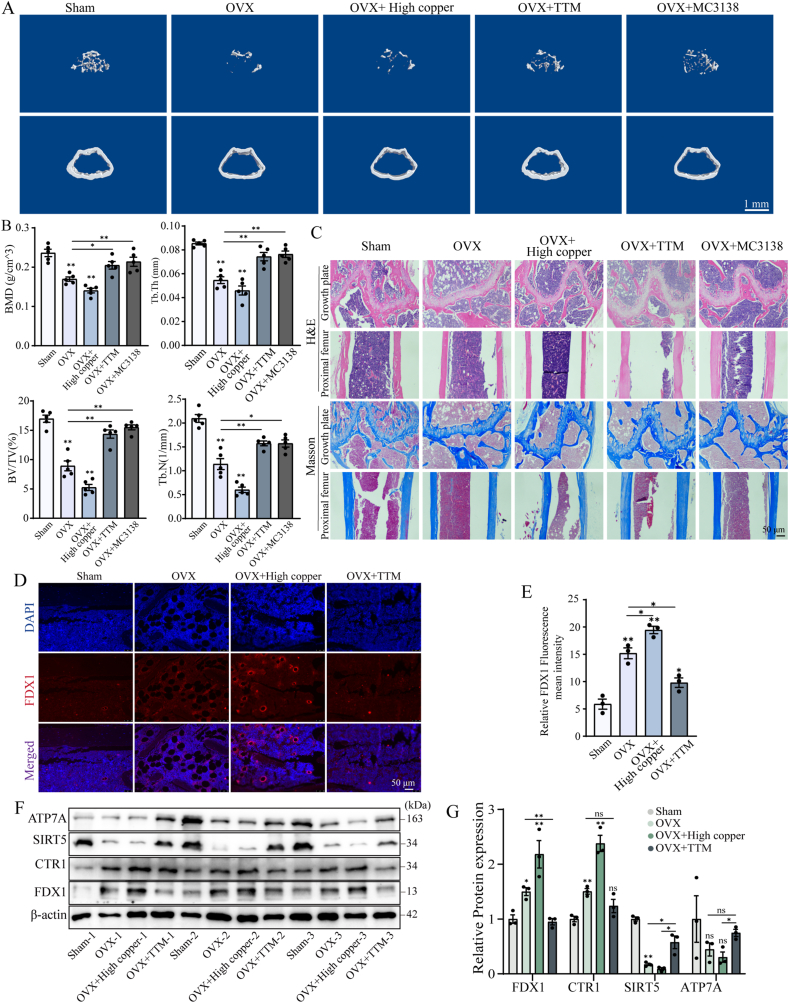
Effects of copper or SIRT5 activator on osteoporosis in ovariectomized mice. **(A)** Micro-CT assay results of the distal femur of mice (scale bar = 1 mm). **(B)** Quantitative results of micro-CT assay show the bone mineral density (BMD), the ratio of the bone volume to the total volume (BV/TV), the number of trabeculae (Tb.N), and the trabecular thickness (Tb.Th) of femoral trabecular bone mass (*n* = 5). **(C)** Representative images of hematoxylin-eosin and Masson’s trichrome staining show the effects of copper, tetrathiomolybdate (TTM, a copper chelator), and/or MC3138 (a SIRT5 activator) on ovariectomy (OVX)-induced bone loss (scale bar = 50 μm). **(D, E)** Representative images of immunofluorescent staining and quantitative results show the effect of copper and/or TTM on FDX1 affected by OVX. **(F, G)** Western blotting assay and quantitative results show the effects of copper or TTM on the protein level of FDX1, SIRT5, CTR1, and ATP7A affected by OVX. ^*ns*^*P* >  0.05, *∗P* < 0.05, and *∗∗P* < 0.01.

To further investigate the effect of cuproptosis or SIRT5 on OVX mice, we next respectively treated OVX mice with high copper, TTM (copper chelator), or MC3138 (SIRT5 activator) (data not shown). The micro-CT analysis results showed that the trabecula was reduced in the OVX group compared with the sham group (Fig. 2A and B). The decrease in trabecula of the OVX group was enhanced by high copper but partially recovered by TTM or MC3138 (Fig. 2A and B). Hematoxylin-eosin staining and Masson trichrome staining showed that bone mass was decreased in the OVX group, which was strengthened by high copper and partially restored by TTM (Fig. 2C). Immunohistochemical staining results showed that the protein level of FDX1 was obviously increased by OVX, which was strengthened by high copper but reduced by TTM (Fig. 2D and E). Further Western blotting results showed that the level of cuproptosis-related proteins was increased in the OVX group, strengthened in the OVX + high copper group, but reduced in the OVX + TTM group, while SIRT5 showed the opposite trend (Fig. 2F and G). These results suggested that estrogen deficiency could potentially trigger cuproptosis, which may partially underlie the development of osteoporosis, while SIRT5 may act as a protective factor during this process.

### Effects of SIRT5 on estrogen-induced osteogenic differentiation in C3H10T1/2 cells

**Figure 3:**
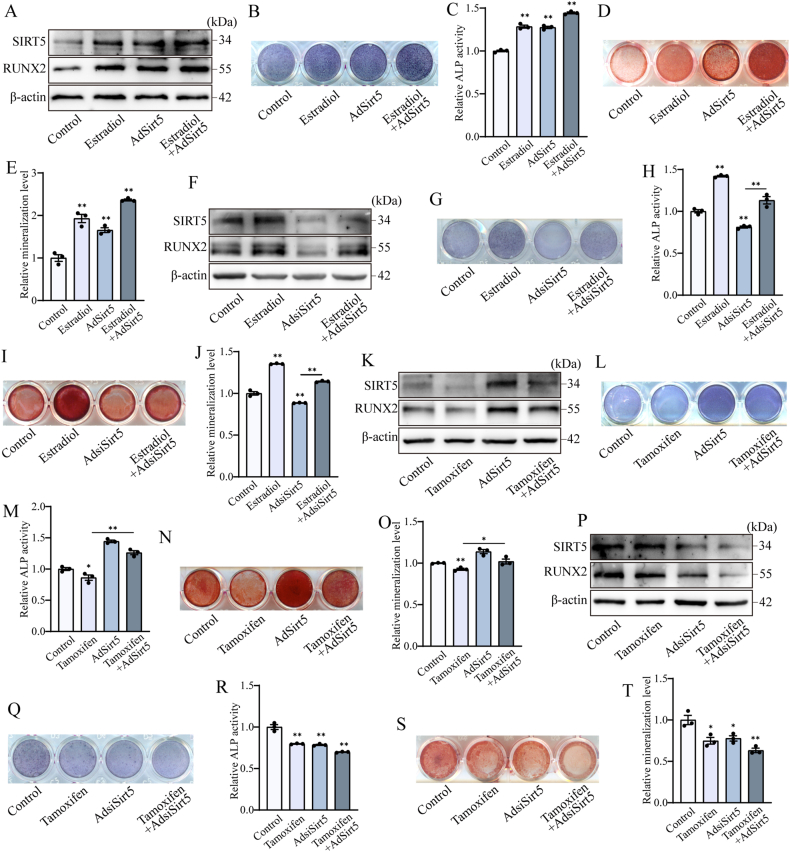
Effects of SIRT5 on estrogen-induced osteogenic differentiation in C3H10T1/2 cells. **(A)** Western blotting assay results show the expression of RUNX2 and SIRT5 in C3H10T1/2 cells treated with estradiol (5 μM) and/or AdSirt5. **(B, C)** Alkaline phosphatase staining and quantitative results show the ALP activity in C3H10T1/2 cells. **(D, E)** Alizarin red S staining and quantitative results show the levels of mineralization. **(F)** Western blotting assay results show the expression of RUNX2 and SIRT5 in C3H10T1/2 cells treated with estradiol (5 μM) and/or AdsiSirt5. **(G, H)** ALP staining and quantitative results show the ALP activity in cells treated with estradiol (5 μM) and/or AdsiSirt5. **(I, J)** Alizarin red S staining and quantitative results show the levels of mineralization in cells treated with estradiol (5 μM) and/or AdsiSirt5. **(K)** Western blotting assay results show the expression of RUNX2 and SIRT5 in cells treated with tamoxifen (5 μM) and/or AdSirt5. **(L, M)** Alkaline phosphatase staining and quantitative results show the ALP activity in cells treated with tamoxifen (5 μM) and/or AdSirt5. **(N, O)** Alizarin red S staining and quantitative results show the levels of mineralization in cells treated with tamoxifen (5 μM) and/or AdSirt5. **(P)** Western blotting assay results show the expression of RUNX2 and SIRT5 in cells treated with tamoxifen (5 μM) and/or AdsiSirt5. **(Q, R)** ALP staining and quantitative results show the ALP activity in cells treated with tamoxifen (5 μM) and/or AdsiSirt5. **(S, T)** Alizarin red S staining and quantitative results show the level of mineralization in cells treated with tamoxifen (5 μM) and/or AdsiSirt5. *∗P* < 0.05 and *∗∗P* < 0.01.

Our previous data demonstrated that SIRT5 activation could improve estrogen deficiency-induced osteoporosis (Fig. 2B). Considering established reports that estrogen promotes osteogenic differentiation of bone marrow mesenchymal stem cells, we next investigated whether SIRT5 participated in mediating estrogen’s pro-osteogenic effects. Results showed that the protein level of RUNX2, ALP activity, and the mineralization level were elevated by estrogen, which was strengthened by SIRT5 (Fig. 3A–E), but weakened by silencing SIRT5 (Fig. 3F–J). To further confirm SIRT5’s role in mediating estrogen’s pro-osteogenic effects, tamoxifen (estrogen receptor antagonist) was used. The results showed that the protein level of RUNX2, ALP activity, and the mineralization level were decreased by tamoxifen, which was partially restored by SIRT5 (Fig. 3K–O), but strengthened by silencing SIRT5 (Fig. 3P–T). These results suggest that SIRT5 can enhance the estrogen-driven promotion of osteogenic differentiation in C3H10T1/2 cells.

### Effects of cuproptosis on osteogenic differentiation in C3H10T1/2 cells

**Figure 4:**
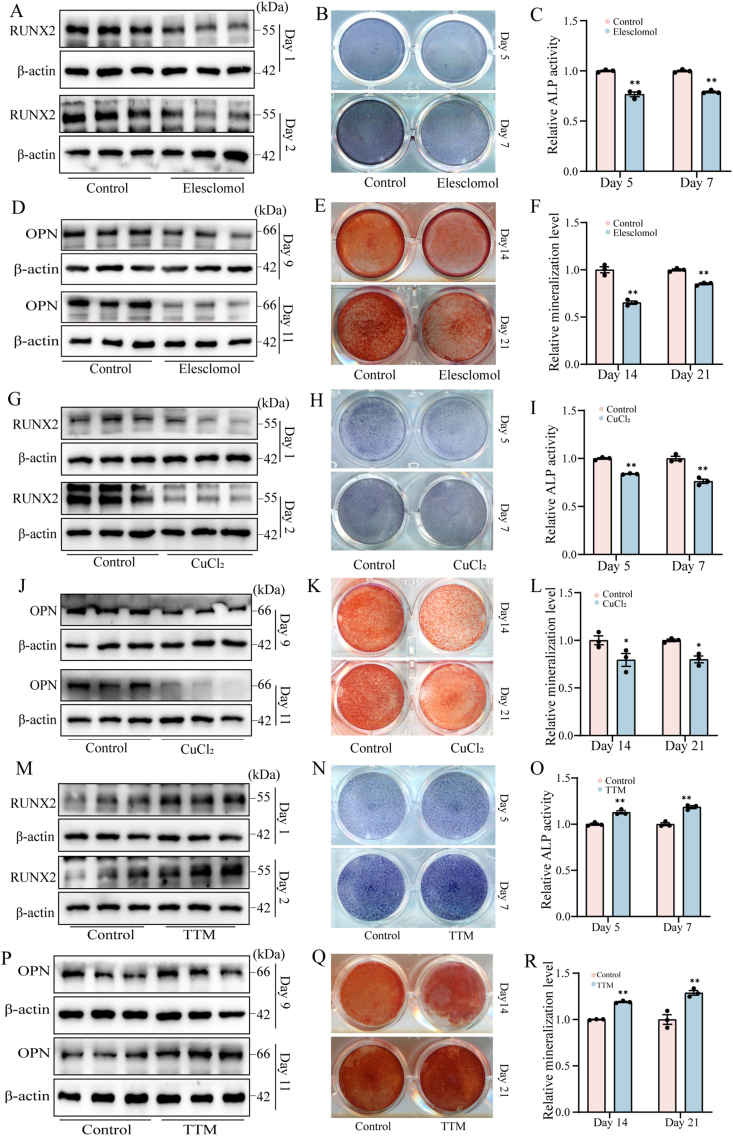
Effects of cuproptosis on osteogenic differentiation in C3H10T1/2 cells. **(A)** Western blotting assay results show the level of RUNX2 in C3H10T1/2 treated with elesclomol (15 nM). **(B, C)** ALP staining and quantitative results show the ALP activities in cells treated with elesclomol (15 nM). **(D)** Western blotting assay results show the level of OPN in C3H10T1/2 treated with elesclomol (15 nM). **(E, F)** Alizarin red S staining and quantitative results show the mineralization level in cells treated with elesclomol (15 nM). **(G)** Western blotting assay results show the level of RUNX2 in C3H10T1/2 treated with CuCl_2_ (5 μM). **(H, I)** ALP staining and quantitative results show the ALP activities in cells treated with CuCl_2_ (5 μM). **(J)** Western blotting assay results show the level of OPN in C3H10T1/2 treated with CuCl_2_ (5 μM). **(K, L)** Alizarin red S staining and quantitative results show the mineralization level in cells treated with CuCl_2_ (5 μM). **(M)** Western blotting assay results show the level of RUNX2 in C3H10T1/2 treated with TTM (10 μM). **(N, O)** ALP staining and quantitative results show the ALP activities in cells treated with Tetrathiomolybdate (TTM, a copper chelator; 10 μM). **(P)** Western blotting assay results show the level of OPN in C3H10T1/2 treated with TTM (10 μM). **(Q, R)** Alizarin red S staining and quantitative results show the mineralization level in cells treated with TTM (10 μM). *∗P* < 0.05 and *∗∗P* < 0.01.

To determine the specific effect of cuproptosis on osteogenic differentiation in C3H10T1/2 cells, we next investigated the effect of elesclomol (cuproptosis inducer), TTM (copper chelator), and/or cupric chloride (CuCl_2_) on the osteogenic differentiation in C3H10T1/2 cells. PCR and Western blotting assay results showed that the protein levels of RUNX2 were reduced by elesclomol (Fig. 4A) but increased by TTM (Fig. 4M). It was confirmed by immunohistochemical staining (data not shown). Histochemical staining results showed that the ALP activities and mineralization were also reduced by elesclomol (Fig. 4B, C, E, F) and but increased by TTM (Fig. 4N, O, Q, R). The protein levels of OPN were reduced by elesclomol (Fig. 4D) but increased by TTM (Fig. 4K). CuCl_2_ showed results similar to elesclomol (Fig. 4G–L). These results suggest that cuproptosis can suppress the osteogenic potential of C3H10T1/2 cells.

### Effects of SIRT5 on the reduced osteogenic potential and cuproptosis induced by elesclomol in C3H10T1/2 cells

**Figure 5:**
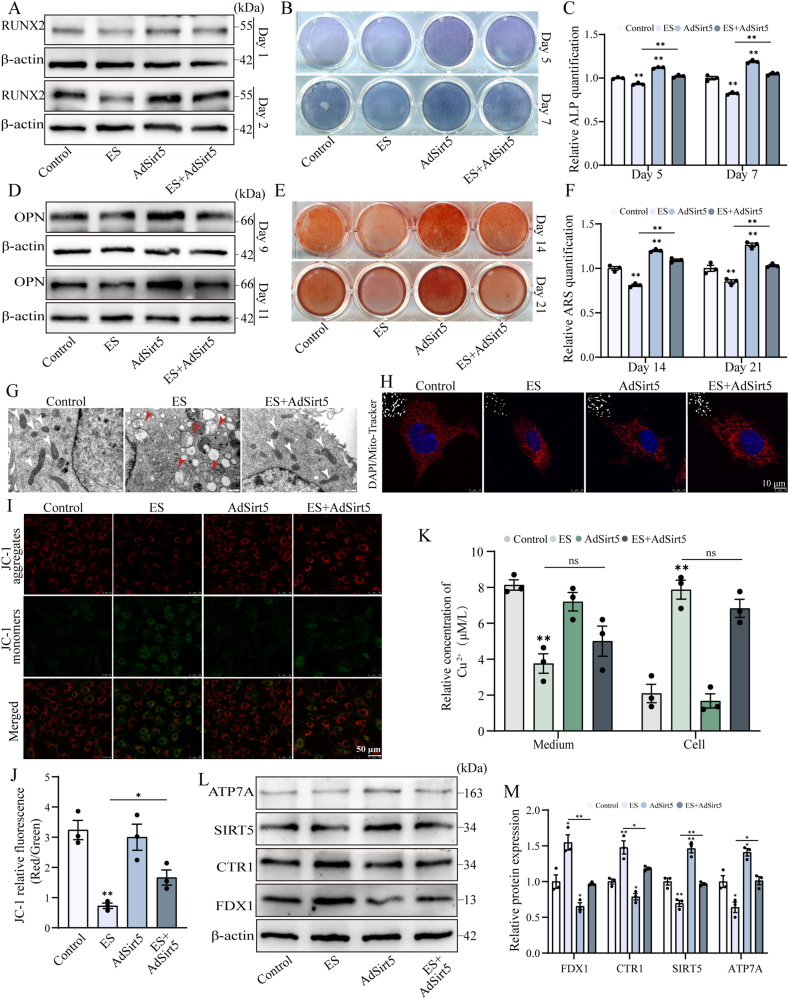
Effects of SIRT5 on the reduced osteogenic potential and cuproptosis induced by elesclomol (ES, 15 nM) in C3H10T1/2 cells. **(A)** Western blotting assay results show the levels of RUNX2 in cells treated with ES and/or AdSirt5. **(B, C)** ALP staining and quantitative results show the ALP activities in cells treated with ES and/or AdSirt5. **(D)** Western blotting assay results show the level of OPN in cells treated with ES and/or AdSirt5. **(E, F)** Alizarin red S staining and quantitative results show the mineralization levels in cells treated with ES and/or AdSirt5. **(G)** Representative transmission electron microscope images show the effect of ES and/or SIRT5 on mitochondria in C3H10T1/2 cells (scale bar = 500 nm). The red arrows indicate the damaged mitochondria, and the white arrows indicate the normal mitochondria. **(H)** Representative immunofluorescent staining images show the effect of ES and/or SIRT5 on mitochondria in C3H10T1/2 cells (scale bar = 10 μm). **(I, J)** JC-1 immunofluorescent assay and quantitative results show the effect of ES and/or SIRT5 on mitochondria in C3H10T1/2 cells (scale bar = 50 μm). **(K)** Copper content assay results show the effect of ES and/or SIRT5 on the medium or cellular level of Cu^2+^. **(L, M)** Western blotting assay and quantitative results show the effect of ES and/or SIRT5 on the level of FDX1, ATP7A, CTR1, and SIRT5 in C3H10T1/2 cells. ^*ns*^*P* > 0.05, *∗P* < 0.05, and *∗∗P* < 0.01.

Our data suggest that SIRT5 can promote osteogenic differentiation in C3H10T1/2 cells, whereas elesclomol (cuproptosis inducer) can inhibit this process. However, whether SIRT5 can rescue the inhibitory effect of elesclomol on osteogenic differentiation remains unknown. Thus, we next determined whether SIRT5 could alleviate the inhibitory effect of elesclomol on osteogenic differentiation. The quantitative PCR and Western blotting results showed that both mRNA and protein levels of RUNX2 and OPN were reduced by elesclomol, which was partially reversed by SIRT5 (Fig. 5A, D) but strengthened by *Sirt5* knockdown (data not shown). The alkaline phosphatase and alizarin red S staining analysis results showed that ALP activity and the mineralization levels were decreased by elesclomol, which was partially reversed by SIRT5 (Fig. 5B, C, E, F) but strengthened by *Sirt5* knockdown (data not shown). These data suggested that SIRT5 may exert its osteopromotioin effects by inhibiting cuproptosis. So, we further analyzed the possible effect of SIRT5 on cuproptosis. The transmission electron microscopy results showed that elesclomol increased vacuoles in the cytoplasm and caused mitochondrial swelling, loss of contents, and broken ridges, which were partly rescued by SIRT5 (Fig. 5G). Mitochondria tracker staining results showed that the fragmented mitochondria induced by elesclomol could be partly reduced by SIRT5 (Fig. 5H). JC-1 fluorescent staining results showed that SIRT5 partially restored the elesclomol-induced reduction of mitochondrial membrane potential (green/red fluorescence ratio) (Fig. 5I and J). SIRT5 cannot obviously affect the intracellular or extracellular copper ion flux (Fig. 5K). The protein levels of cuproptosis markers, such as CTR1 and FDX1, were increased by elesclomol, which were reduced by SIRT5 (Fig. 5L and M). These data suggest that SIRT5 may partially alleviate the reduced osteogenic differentiation and cuproptosis induced by elesclomol.

### Effects of FDX1 on SIRT5-mediated reversal of elesclomol-induced osteogenic potential reduction and cuproptosis in C3H10T1/2 cells

**Figure 6:**
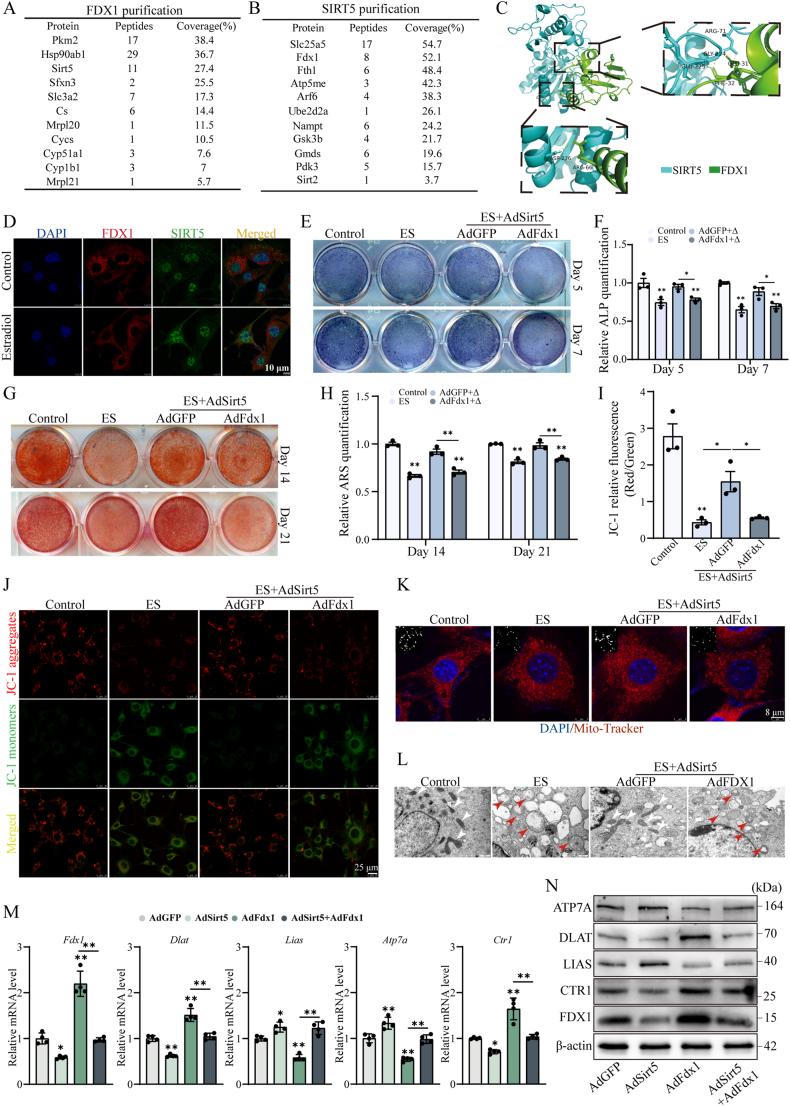
Effects of FDX1 on SIRT5-mediated reversal of elesclomol (ES; 15 nM)-induced osteogenic potential reduction and cuproptosis in C3H10T1/2 cells. **(A)** Mass spectrometry of FDX1 purification beads show the interacted proteins. **(B)** Mass spectrometry of SIRT5 purification beads show the interacted proteins. **(C)** Molecular docking analysis results show the interaction between SIRT5 and FDX1. FDX1 is represented as a cyan cartoon, SIRT5 is presented as a pistachio cartoon, and their binding sites are displayed. **(D)** Immunofluorescent staining results show the colocalization of SIRT5 and FDX1 in C3H10T1/2 cells treated with estradiol (scale bar = 10 μm). **(E, F)** ALP staining and quantitative results show the ALP activities in cells treated with ES, AdSirt5, and/or AdFDX1. **(G, H)** Alizarin red S staining and quantitative results show the mineralization levels in cells treated with ES, AdSirt5, and/or AdFDX1. **(I, J)** JC-1 immunofluorescent staining and quantitative results show the changes of membrane potential in cells treated with ES, AdSirt5, and/or AdFDX1 (scale bar = 25 μm)**. (K)** Immunofluorescent staining results show the morphology of mitochondria in C3H10T1/2 cells treated with ES, AdSirt5, and/or AdFDX1 (scale bar = 8 μm). **(L)** Representative transmission electron microscopy images show the morphology of mitochondria in C3H10T1/2 cells treated with ES, AdSirt5, and/or AdFDX1 (scale bar = 500 nm). The red arrows indicate injuried mitochondria, and the white arrows indicate normal mitochondria. **(M)** Real-time PCR assay results show the effect of SIRT5 and/or FDX1 on the mRNA expression level of cuproptosis markers. **(N)** Western blotting assay results show the effect of SIRT5 and/or FDX1 on the level of cuproptosis markers. *∗P* < 0.05 and *∗∗P* < 0.01.

Previous data suggest that the inhibitory effect of SIRT5 on cuproptosis may be associated with down-regulation of FDX1. We then determined the possible mechanism through which SIRT5 carries out this function. Mass spectrometry analysis and molecular docking analysis results showed that there may exist an interaction between SIRT5 and FDX1 (Fig. 6A–C), which was confirmed by co-immunoprecipitation assays (data not shown). The immunofluorescent assay results further showed that the location of SIRT5 was similar to that of FDX1 in the cytoplasm (Fig. 6D). To investigate whether FDX1 could mitigate the effect of SIRT5 on elesclomol-reduced osteogenic potential and cuproptosis, the recombinant adenovirus for *Fdx1* was constructed and functionally validated (data not shown). The data showed that SIRT5 partially alleviated the inhibitory effect of elesclomol on osteogenic differentiation, which was almost abolished by FDX1, and reduced RUNX2, OPN (data not shown), ALP activity, and mineralization levels (Fig. 6E–H). Besides, JC-1 fluorescent staining results showed that SIRT5 partially restored the elesclomol-induced decrease of mitochondrial membrane potential (green/red fluorescence ratio), which was almost abolished by FDX1 (Fig. 6I and J). The mitochondria tracker staining and transmission electron microscopy assay results showed that the SIRT5-mediated restoration of mitochondrial morphology was almost cancelled by FDX1 (Fig. 6K and L). The real-time PCR assay results showed that the mRNA expression level of *Fdx1*, *Dlat*, and *Ctrl* were reduced by *Sirt5* overexpression, while *Lias* and *Atp7a* were increased; these effects were abrogated by *Fdx1* overexpression obviously (Fig. 6M). The effects of SIRT5 on reducing the protein level of CTR1 and FDX1 were diminished by *Fdx1* overexpression (Fig. 6N). These results suggest that the reversal effects of SIRT5 on elesclomol-induced osteogenic potential reduction and cuproptosis can be abolished by FDX1.

### Effects of SIRT5 and estrogen on malonylation modification and protein level of FDX1 in C3H10T1/2 cells

**Figure 7:**
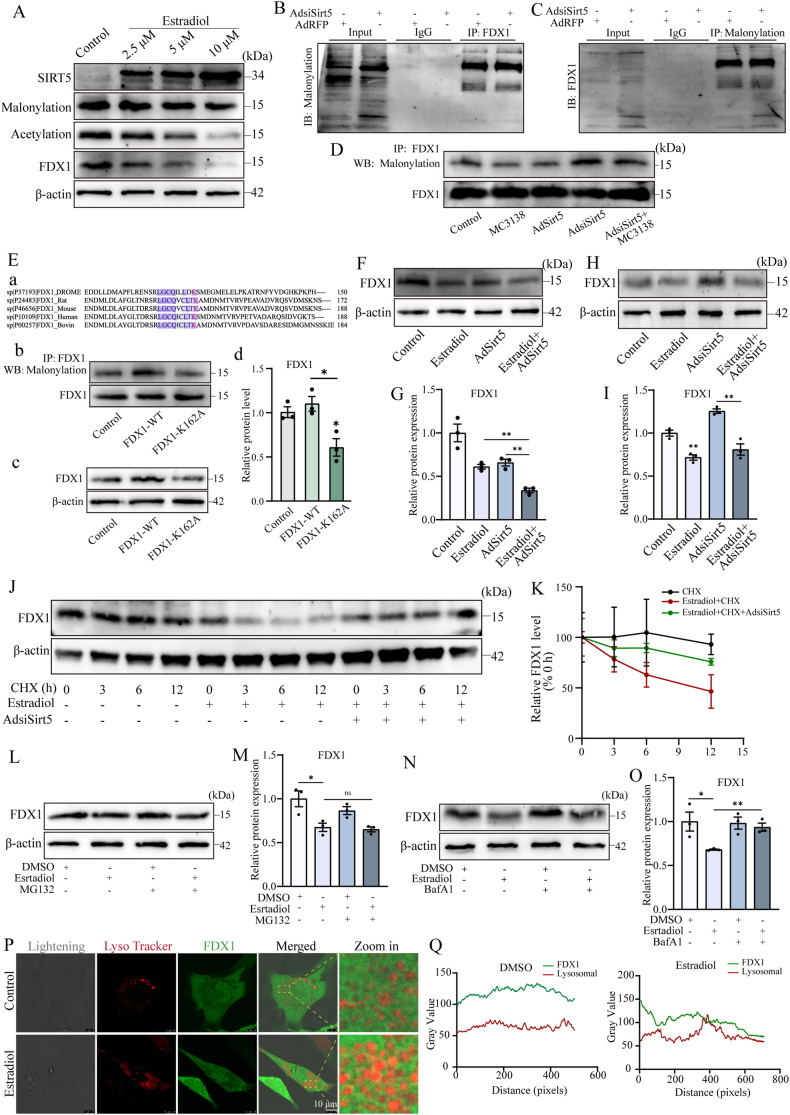
Effects of SIRT5 and estrogen on malonylation modification and protein level of FDX1 in C3H10T1/2 cells. **(A)** Western blotting assay results show the levels of SIRT5, FDX1, malonylation, and acetylation in C3H10T1/2 cells treated with estradiol (2.5 μM, 5 μM, 10 μM). **(B, C)** Co-immunoprecipitation assay results show the malonylation modification of FDX1 and in C3H10T1/2 cells transfected with Sirt5 knockdown. **(D)** Co-immunoprecipitation and Western blotting assay results show the effects of SIRT5, *Sirt5* knockdown, and/or MC3138 on FDX1 malonylation modification. **(E)** Site specific mutation, co-immunoprecipitation, and Western blotting assay results show that lysine 162 of FDX1 is critical for its malonylation modification and protein level (a. Amino acid sequence alignment of FDX1 proteins across diverse species; b. Co-immunoprecipitation and Western blotting assay results show the effect of FDX1 K162A mutation on its malonylation modification; c. Western blotting assay results show the effect of FDX1 K162A mutation on its protein level; d. Quantitative results of Western blotting assay show the effect of FDX1 K162A mutation on its protein level). **(F, G)** Western blotting assay and quantitative results show levels of FDX1 in C3H10T1/2 cells treated with estradiol (5 μM) and/or AdSirt5. **(H, I)** Representative Western blotting images of FDX1 in C3H10T1/2 cells treated with estradiol (5 μM) and/or Sirt5 knockdown. **(J, K)** Western blotting assay and quantitative results show the levels of FDX1 in C3H10T1/2 treated with CHX (100 μM), CHX with estradiol (5 μM), or CHX with estradiol and AdsiSirt5. **(L, M)** Western blotting assay and quantitative results show the levels of FDX1 in C3H10T1/2 cells treated with MG132 (10 μM) and/or estradiol (5 μM). **(N, O)** Western blotting assay and quantitative results show the levels of FDX1 in C3H10T1/2 cells treated with BafA1 (100 nM) and/or estradiol (5 μM). **(P, Q)** Immunofluorescent staining results show the colocalization of FDX1 and Lyso-tracker in C3H10T1/2 cells treated with estradiol (5 μM). CHX: Cycloheximide (100 μM); MG132: Z-Leu-Leu-Leu-al (10 μM); BafA1: Bafilomycin A1 (100 nM); DAPI: 4′,6-diamidino-2-phenylindole; MC3138: SIRT5 activator. *∗P* < 0.05 and *∗∗P* < 0.01.

Our data have demonstrated that estrogen can increase the mRNA level of Fdx1 but reduce its protein level, which suggests that estrogen may simultaneously regulate FDX1 stability through post-transcriptional mechanisms. Given that SIRT5 has been reported to participate in various protein modifications, including demalonylation and deacetylation. Thus, we investigated whether estrogen could affect FDX1 post-translational modifications via SIRT5. Western blotting analysis revealed that SIRT5 was elevated, while FDX1, acetylation, and malonylation levels were decreased by estrogen (Fig. 7A), which was further confirmed by immunofluorescent assay (data not shown). Co-immunoprecipitation analysis results further demonstrated that *Sirt5* knockdown increased the malonylation level of FDX1 (Fig. 7B and C), whereas the acetylation level of FDX1 obviously remained unchanged (data not shown). FDX1 malonylation modification level was increased by *Sirt5* knockdown, which was notably reduced by SIRT5 activator (Fig. 7D). Site-specific mutation, co-immunoprecipitation, and Western blotting assay results showed that lysine 162 (K162) of FDX1 is critical for its malonylation modification (Fig. 7E).

To determine whether estrogen can affect the stability of FDX1 through SIRT5, we treated cells with cycloheximide (the protein synthesis inhibitor), estrogen, and *Sirt5* knockdown. Western blotting assay results showed that estrogen could reduce the protein level of FDX1 and increase the degradation rate of FDX1, which was partly reversed by silencing SIRT5 (Fig. 7F–K). We then determined how estrogen regulated the degradation of FDX1, and the results showed that BafA1 (the inhibitor of lysosome) significantly reversed the estrogen-induced decrease of FDX1 protein level (Fig. 7N and O), whereas MG132 (the inhibitor of proteasome) did not show the same effect (Fig. 7L and M). Further immunofluorescent staining results showed that the colocalization of FDX1 and lysosome was increased in cells treated with estrogen compared with the control group (Fig. 7P and Q). These results suggest that SIRT5 may regulate FDX1 degradation through the lysosomal pathway by mediating its demalonylation modification.

## Discussion

Osteoporosis represents a prevalent condition among aging populations 1, 2, 3. Impaired osteoblast activity linked to estrogen deficiency is recognized as a key factor contributing to the reduced bone formation.^3^ Drugs that act as agonists of sirtuins hold clinical promise in the treatment of age-related diseases, including osteoporosis.^9^ In this study, we identify that SIRT5 may act as an effective estrogen-responsive mediator to promote bone formation, potentially through the Estrogen/SIRT5/FDX1 axis to mitigate cuproptosis in mesenchymal stem cells. Our findings suggest SIRT5 could serve as a potential target to improve estrogen deficiency-associated osteoporosis.

SIRT5, a member of the sirtuin family, has been identified as a mitochondrial lysine deacylase with implications in various physiological processes; the SIRT5 expression has been associated with bone loss.^41^ Reports and our prior work indicate that SIRT5 is involved in promoting osteogenic differentiation, and its expression was reduced in bone loss models.^20^^,^^42^ To date, the precise mechanistic relationship between estrogen deficiency-induced osteoporosis and SIRT5 remains incompletely characterized. In this study, we found that SIRT5 expression was significantly down-regulated in OVX mice. Conversely, estrogen treatment resulted in concentration-dependent up-regulation of SIRT5 in C3H10T1/2 cells. Further experiments demonstrated that SIRT5 enhanced the osteogenic potential of estrogen. while Sirt5 silencing attenuated this effect. Administration of MC3138, a SIRT5 activator, could significantly improve the OVX-induced osteoporosis. These data suggest that SIRT5 may participate in mediating the effect of estrogen on promoting osteogenic differentiation.

Dysregulation of copper homeostasis has been linked to age-related bone loss 43, 44, 45. While low copper concentrations promote osteoblast proliferation and differentiation, elevated levels exert cytotoxic effects.^30^ Cuproptosis, a recently described form of cell death caused by copper overload and impaired protein lipoylation, provides novel insights into how copper imbalance may contribute to bone disorders.^32^ However, the precise contribution of copper to osteoporosis pathogenesis remains unclear. Our findings revealed decreased serum copper levels but increased femoral copper concentrations in OVX mice, consistent with clinical observations of lower serum copper in postmenopausal women with osteoporosis.^46^ This pattern indicates a localized, bone-selective copper dysregulation rather than systemic overload, with the accumulated copper potentially initiating cuproptosis. Correspondingly, cuproptosis-related markers, including FDX1 and CTR1, were up-regulated in OVX mice, while ATP7A expression was decreased; these changes were partially reversed by TTM, a copper chelator. *In vivo*, TTM treatment markedly attenuated OVX-induced bone loss, whereas dietary copper excess exacerbated osteoporotic phenotypes. In C3H10T1/2 cells, cuproptosis inducers (elesclomol or CuCl_2_) impaired osteogenesis, which was almost abrogated by TTM co-treatment. These data support cuproptosis as a novel mechanistic component in osteoporosis. Notably, SIRT5 overexpression partially rescued the elesclomol-induced suppression of osteogenesis, while SIRT5 knockdown intensified this effect. Elesclomol exposure up-regulated FDX1 and induced mitochondrial dysfunction (structural abnormalities and depolarization), with SIRT5 exhibiting partial protective effects. These results suggest that SIRT5 may confer resistance to cuproptosis.

FDX1 plays a central role in cuproptosis by reducing Cu^2+^, which triggers toxic aggregation of mitochondrial proteins and subsequent cell death.^32^^,^^36^ Elevated FDX1 expression has been reported in multiple degenerative disorders.^37^^,^^38^^,^^47^ In our study, OVX mice exhibited increased FDX1 expression, while estrogen treatment reduced FDX1 protein levels in C3H10T1/2 cells, despite a paradoxical increase in FDX1 mRNA. This striking disconnect strongly suggests the presence of complex post-transcriptional regulation of FDX1. Using mass spectrometry and molecular docking, we identified a direct interaction between SIRT5 and FDX1, which was subsequently validated by co-immunoprecipitation and immunofluorescence assays. Functionally, FDX1 overexpression abrogated SIRT5’s protective effects against elesclomol-induced cuproptosis and osteogenic impairment, establishing FDX1 as a key downstream target of SIRT5 in bone homeostasis. Mechanistically, estrogen up-regulated SIRT5, which in turn reduced both FDX1 protein levels and malonylation modifications. SIRT5 knockdown specifically elevated FDX1 malonylation without affecting acetylation, identifying demalonylation as the critical regulatory modification. Estrogen accelerated FDX1 degradation, an effect attenuated by SIRT5 silencing. We further demonstrated estrogen-induced FDX1 degradation through lysosomal pathways. This conclusion is supported by two key findings: The lysosome inhibitor BafA1, but not the proteasome inhibitor MG132, prevented estrogen-mediated FDX1 reduction; and estrogen treatment significantly enhanced FDX1-lysosome colocalization. This lysosomal regulation, combined with SIRT5-dependent FDX1 demalonylation, constitutes a novel dual mechanism through which the Estrogen/SIRT5/FDX1 axis coordinates cuproptosis suppression and osteogenic promotion.

Collectively, our results suggest that estrogen deficiency-associated osteoporosis may be partly due to the suppressed SIRT5 expression and bone-specific copper dysregulation, which together promote cuproptosis. SIRT5 mediates estrogen’s anabolic effects in bone by alleviating cuproptosis through two coordinated actions: facilitating FDX1 demalonylation and enhancing lysosomal degradation of FDX1. These findings advance our understanding of the pathogenesis of estrogen deficiency-induced osteoporosis and highlight that SIRT5 may be an attractive therapeutic candidate target for the management of postmenopausal osteoporosis.

## CRediT authorship contribution statement

**Fanglin Ye:** Writing – original draft, Methodology, Investigation, Formal analysis, Data curation. **Dongmei He:** Methodology, Investigation, Formal analysis, Data curation. **Wenting Liu:** Validation, Methodology, Investigation. **Jie Cai:** Visualization, Methodology, Investigation, Formal analysis, Data curation. **Aihua Ye:** Resources, Methodology. **Zhenghao Xu:** Resources, Methodology. **Wenge He:** Resources, Methodology, Conceptualization. **Yuxi Su:** Project administration, Conceptualization. **Junyi Liao:** Funding acquisition. **Baicheng He:** Writing – review & editing, Writing – original draft, Supervision, Resources, Project administration, Funding acquisition.

## Ethics declaration

The animal experimentation was approved by the Medical Research Ethics Committee of Chongqing Medical University.

## Data availability

The datasets employed within this work are made available upon request and directed to the corresponding author.

## Funding

The research was financially supported by the Chongqing Medical University Program for Youth Innovation in Future Medicine (China) (No. W0154 to B.C.H. and J.Y.L.) and 10.13039/501100002865Chongqing Science and Technology Bureau (China) (No. CSTB2024NSCQ-MSX0411 to B.C.H.).

## Conflict of interests

The authors declared no conflict of interests.

## References

[1] Jang B., Kim Y., Song J., Kim Y.W., Lee W.Y. (2024). Identifying herbal candidates and active ingredients against postmenopausal osteoporosis using biased random walk on a multiscale network. Int J Mol Sci.

[2] Lanzolla G., Sabini E., Beigel K. (2024). Pharmacological inhibition of HIF2 protects against bone loss in an experimental model of estrogen deficiency. Proc Natl Acad Sci U S A.

[3] Khosla S., Monroe D.G. (2018). Regulation of bone metabolism by sex steroids. Cold Spring Harb Perspect Med.

[4] Jiang Y., Horkeby K., Henning P. (2025). Membrane-initiated estrogen receptor-α signaling in osteoblasts is crucial for normal regulation of the cortical bone in female mice. Bone Res.

[5] Khosla S., Oursler M.J., Monroe D.G. (2012). Estrogen and the skeleton. Trends Endocrinol Metab.

[6] Nilsson S., Mäkelä S., Treuter E. (2001). Mechanisms of estrogen action. Physiol Rev.

[7] Hsu S.H., Chen L.R., Chen K.H. (2024). Primary osteoporosis induced by androgen and estrogen deficiency: the molecular and cellular perspective on pathophysiological mechanisms and treatments. Int J Mol Sci.

[8] Morris B.J. (2013). Seven sirtuins for seven deadly diseases of aging. Free Radic Biol Med.

[9] Li Q., Cheng J.C., Jiang Q., Lee W.Y. (2021). Role of sirtuins in bone biology: potential implications for novel therapeutic strategies for osteoporosis. Aging Cell.

[10] Zhang T., Wang L., Duan X. (2024). Sirtuins mediate mitochondrial quality control mechanisms: a novel therapeutic target for osteoporosis. Front Endocrinol.

[11] Waykar T.R., Mandlik S.K., Mandlik D.S. (2024). Sirtuins: exploring next-gen therapeutics in the pathogenesis osteoporosis and associated diseases. Immunopharmacol Immunotoxicol.

[12] Artsi H., Cohen-Kfir E., Gurt I. (2014). The Sirtuin1 activator SRT3025 down-regulates sclerostin and rescues ovariectomy-induced bone loss and biomechanical deterioration in female mice. Endocrinology.

[13] Chen E.E.M., Zhang W., Ye C.C.Y. (2017). Knockdown of SIRT7 enhances the osteogenic differentiation of human bone marrow mesenchymal stem cells partly via activation of the Wnt/β-catenin signaling pathway. Cell Death Dis.

[14] Chen S., Jin J., Xu Z., Han H., Wu L., Li Z. (2024). Catalpol attenuates osteoporosis in ovariectomized rats through promoting osteoclast apoptosis via the Sirt6-ERα-FasL axis. Phytomedicine.

[15] Qin Y., Hu C., Jin J. (2024). Bilobalide ameliorates osteoporosis by influencing the SIRT3/NF-κB axis in osteoclasts and promoting M2 polarization in macrophages. Int J Biol Macromol.

[16] Fiorentino F., Castiello C., Mai A., Rotili D. (2022). Therapeutic potential and activity modulation of the protein lysine deacylase sirtuin 5. J Med Chem.

[17] Wang Y., Chen H., Zha X. (2022). Overview of SIRT5 as a potential therapeutic target: structure, function and inhibitors. Eur J Med Chem.

[18] Mao J., Wang D., Wang D. (2023). SIRT5-related desuccinylation modification of AIFM1 protects against compression-induced intervertebral disc degeneration by regulating mitochondrial homeostasis. Exp Mol Med.

[19] Zhang Y., Wang J., Luan J., Liu C., Cui Y., Han J. (2024). Sirt5 desuccinylates Cdc42 to mediate osteoclastogenesis and bone remodeling in mice. Genes Dis.

[20] Liu L., Ye F., Jiang Y. (2025). SIRT5 promotes the osteo-inductive potential of BMP9 by stabilizing the HIF-1α protein in mouse embryonic fibroblasts. Genes Dis.

[21] Doguer C., Ha J.H., Collins J.F. (2018). Intersection of iron and copper metabolism in the mammalian intestine and liver. Compr Physiol.

[22] Teschke R., Eickhoff A. (2024). Wilson disease: Copper-mediated cuproptosis, iron-related ferroptosis, and clinical highlights, with comprehensive and critical analysis update. Int J Mol Sci.

[23] Lutsenko S. (2010). Human copper homeostasis: a network of interconnected pathways. Curr Opin Chem Biol.

[24] Sierpinska T., Konstantynowicz J., Orywal K., Golebiewska M., Szmitkowski M. (2014). Copper deficit as a potential pathogenic factor of reduced bone mineral density and severe tooth wear. Osteoporos Int.

[25] Rondanelli M., Faliva M.A., Infantino V. (2021). Copper as dietary supplement for bone metabolism: a review. Nutrients.

[26] Zhang Z., Tang H., Du T., Yang D. (2024). The impact of copper on bone metabolism. J Orthop Transl.

[27] Yang L., Perez-Amodio S., Barrère-de Groot F.Y.F., Everts V., van Blitterswijk C.A., Habibovic P. (2010). The effects of inorganic additives to calcium phosphate on *in vitro* behavior of osteoblasts and osteoclasts. Biomaterials.

[28] Ren L., Wong H.M., Yan C.H., Yeung K.W.K., Yang K. (2015). Osteogenic ability of Cu-bearing stainless steel. J Biomed Mater Res B Appl Biomater.

[29] Zhang X., Liu H., Li L. (2022). Promoting osteointegration effect of Cu-alloyed titanium in ovariectomized rats. Regen Biomater.

[30] Bane T., Siegel L., Bertels J., Ratz K., Rubessa M., Wheeler M. (2018). 208 the effect of copper on the differentiation of adipose-derived stem cells into osteoblasts. Reprod Fertil Dev.

[31] Qi Y., Wang H., Chen X., Zhu Y. (2021). The role of TGF-β1/Smad3 signaling pathway and oxidative stress in the inhibition of osteoblast mineralization by copper chloride. Environ Toxicol Pharmacol.

[32] Tsvetkov P., Coy S., Petrova B. (2022). Copper induces cell death by targeting lipoylated TCA cycle proteins. Science.

[33] Han J., Luo J., Wang C., Kapilevich L., Zhang X.A. (2024). Roles and mechanisms of copper homeostasis and cuproptosis in osteoarticular diseases. Biomed Pharmacother.

[34] Sun Y., Chen P., Zhao B. (2024). Role of extracellular vesicles associated with microRNAs and their interplay with cuproptosis in osteoporosis. Noncoding RNA Res.

[35] Xie C., Sun Q., Chen J. (2024). Cu-*Tremella fuciformis* polysaccharide-based tumor microenvironment-responsive injectable gels for cuproptosis-based synergistic osteosarcoma therapy. Int J Biol Macromol.

[36] Zulkifli M., Spelbring A.N., Zhang Y. (2023). FDX1-dependent and independent mechanisms of elesclomol-mediated intracellular copper delivery. Proc Natl Acad Sci U S A.

[37] Cui Y., Chen Y., Gan N. (2023). A novel cuproptosis-related diagnostic gene signature and differential expression validation in atherosclerosis. Mol Biomed.

[38] Chen X., Li K., Xiao Y. (2024). SP1/CTR1-mediated oxidative stress-induced cuproptosis in intervertebral disc degeneration. Biofactors.

[39] Hu T., Shukla S.K., Vernucci E. (2021). Metabolic rewiring by loss of Sirt5 promotes Kras-induced pancreatic cancer progression. Gastroenterology.

[40] Ambi A., Stanisavljevic A., Victor T.W. (2023). Evaluation of copper chelation therapy in a transgenic rat model of cerebral amyloid angiopathy. ACS Chem Neurosci.

[41] Avilkina V., Chauveau C., Ghali Mhenni O. (2022). Sirtuin function and metabolism: role in pancreas, liver, and adipose tissue and their crosstalk impacting bone homeostasis. Bone.

[42] Ke K.X., Gao X., Liu L. (2024). Leptin attenuates the osteogenic induction potential of BMP9 by increasing β-catenin malonylation modification via Sirt5 down-regulation. Aging.

[43] Bhardwaj P., Rai D.V., Garg M.L. (2013). Zinc as a nutritional approach to bone loss prevention in an ovariectomized rat model. Menopause.

[44] Bizoń A., Słowiak A., Franik G., Biernacka-Bartnik A., Madej P. (2020). Zinc, copper, sirtuin 1 concentration, and glucose metabolism parameters in the blood of women with polycystic ovary syndrome. Gynecol Endocrinol.

[45] Vaquero M.P., García-Maldonado E., Gallego-Narbón A., Zapatera B., Alcorta A., Martínez-Suárez M. (2024). Iron deficiency is associated with elevated parathormone levels, low vitamin D status, and risk of bone loss in omnivores and plant-based diet consumers. Int J Mol Sci.

[46] Okyay E., Ertugrul C., Acar B., Sisman A.R., Onvural B., Ozaksoy D. (2013). Comparative evaluation of serum levels of main minerals and postmenopausal osteoporosis. Maturitas.

[47] Zhou Y., Li X., Ng L. (2023). Identification of copper death-associated molecular clusters and immunological profiles in rheumatoid arthritis. Front Immunol.

